# Improving the outcome of kidney transplantation by ameliorating renal ischemia reperfusion injury: lost in translation?

**DOI:** 10.1186/s12967-016-0767-2

**Published:** 2016-01-20

**Authors:** T. C. Saat, E. K. van den Akker, J. N. M. IJzermans, F. J. M. F. Dor, R. W. F. de Bruin

**Affiliations:** Department of Surgery, Erasmus MC, University Medical Center, Room Ee-100, Dr. Molewaterplein 50, 3015 GE Rotterdam, The Netherlands

**Keywords:** Kidney transplantation, Ischemia reperfusion injury, Translation, Treatment

## Abstract

Kidney transplantation is the treatment of choice in patients with end stage renal disease. During kidney transplantation ischemia reperfusion injury (IRI) occurs, which is a risk factor for acute kidney injury, delayed graft function and acute and chronic rejection. Kidneys from living donors show a superior short- and long-term graft survival compared with deceased donors. However, the shortage of donor kidneys has resulted in expansion of the donor pool by using not only living- and brain death donors but also kidneys from donation after circulatory death and from extended criteria donors. These grafts are associated with an increased sensitivity to IRI and decreased graft outcome due to prolonged ischemia and donor comorbidity. Therefore, preventing or ameliorating IRI may improve graft survival. Animal experiments focus on understanding the mechanism behind IRI and try to find methods to minimize IRI either before, during or after ischemia. This review evaluates the different experimental strategies that have been investigated to prevent or ameliorate renal IRI. In addition, we review the current state of translation to the clinical setting. Experimental research has contributed to the development of strategies to prevent or ameliorate IRI, but promising results in animal studies have not yet been successfully translated to clinical use.

## Background

Kidney transplantation is the treatment of choice in patients with end stage renal disease. Increased prevalence of end stage renal disease, and improved results after kidney transplantation have contributed to the increased shortage of donor organs and the need to expand the donor pool [1, 2]. Organs from living donors have a superior graft survival compared with deceased donors [3, 4]. The superior outcome of living donors kidneys is associated with shorter warm and cold ischemia, shorter waiting time for the recipient and ‘healthier’ donor kidneys [5]. Warm ischemia occurs after the blood supply has been cut off while the organ is still in the donor. During storage of the transplant, the temperature is reduced to approximately 4 °C. During this cold ischemia period, metabolism is significantly reduced which allows for prolonged preservation of the organ until transplantation.

To bridge the growing gap between organ demand and supply, donation after circulatory death (DCD) donors [6] and extended criteria donors are increasingly being used [3–5]. Donation after brain death (DBD) donors are exposed to physiological changes during brain death, which may lead to organ damage and inferior graft survival compared to living donors [7, 8]. DCD donors do not develop the physiological changes of DBD donors, but suffer from prolonged warm ischemia times during cardiac arrest. DCD kidneys have an increased incidence of delayed graft function (DGF) of 73 % compared to 27 % in DBD donor kidneys [9], while the rate of acute rejection is similar in both. Despite the higher incidence of DGF, DCD kidneys show no differences in long-term graft survival compared with DBD kidneys [6, 10].

Although the use of DCD donors has been increased, the total number of cadaveric donors remains stable, while the waiting list continues to grow [6, 11]. Therefore, the number of extended criteria donors is increasing. Extended criteria donors are defined as donors being >60 years old, or aged >50–59 years old with ≥2 of the following risk factors: history of hypertension, serum creatinine level ≥1.5 mg/dL, or death resulting from a cerebrovascular accident [12, 13]. Organs from extended criteria donors are associated with a higher incidence of DGF, lower graft survival and suboptimal kidney function [13, 14]. Recipients of kidneys procured from extended criteria donors show a 1.7-fold greater risk of graft lost compared to recipients with a kidney from an ‘ideal donor’ (10–39 years old without hypertension or stroke as a cause of death and a serum creatinine concentration <1.5 mg/dL) [15].

Ischemia reperfusion injury (IRI) is an inevitable consequence of kidney transplantation and has major consequences for graft- and patient survival [16–18]. Renal IRI is a known risk factor for DGF [19], acute kidney injury [20] and acute and chronic rejection [21]. Donor type is strongly associated with the severity of renal IRI [22]. DCD donors and extended criteria donors are more vulnerable to IRI since donor kidneys suffer from prolonged warm ischemia time, increased donor age or comorbidity of the donor [13, 14]. Prevention or reduction of IRI could improve graft survival and decrease patient morbidity.

### Renal ischemia reperfusion injury

Renal IRI is unavoidable during transplantation and is a risk factor for DGF [19], acute kidney injury [18, 23] and acute and chronic rejection [24, 25]. Acute kidney injury is associated with high morbidity, prolonged hospitalization, and increased mortality [20, 26]. During ischemia there is a lack of O_2_ and nutrients, which results in a decrease of oxidative metabolism, accumulation of metabolic waste products and depletion of ATP [19, 20].

Reperfusion leads to rewarming, reoxygenation and a return to aerobic metabolism. However, reactive oxygen species are generated which directly injure the cytoskeletal and functional cellular components [19]. Normally, antioxidant enzymes may counteract the effects of reactive oxygen species, but their protective effect is overwhelmed by the rapid production of reactive oxygen species, resulting in tissue injury and cell death [27].

During reperfusion, tissue injury is exacerbated by an inflammatory response, which initiates a cascade of deleterious cellular responses [18, 19]. Inflammatory cytokines are up regulated, and chemokines and complement are released, which results in the migration and activation of leukocytes.

The mechanism underlying IRI is multifactorial. Due to its complexity, IRI provides different targets to prevent or ameliorate renal IRI before, during or after transplantation [23].

### Strategies to ameliorate renal IRI

Strategies to reduce renal IRI can be implemented in both donor and recipient, and before, during and after transplantation. Treatment of IRI can be focused on scavenging reactive oxygen species, reducing inflammation, stimulating cell survival and regeneration, or a combination thereof. Prevention of ischemia is impossible by inducing resistance against ischemia before organ retrieval. Pre-treatment of living donors is feasible, provided it does not affect the health and wellbeing of the donor. In post-mortem donors the situation is more difficult since these donors are not able to give informed consent and ethical issues may rise. During preservation treatment is possible by using machine preservation and/or by adding protective agents to the perfusion fluid, pre- or post-conditioning is feasible during transplantation. After transplantation, treating the recipient, after informed consent, may reduce the damage caused by IRI.

**Fig. 1:**
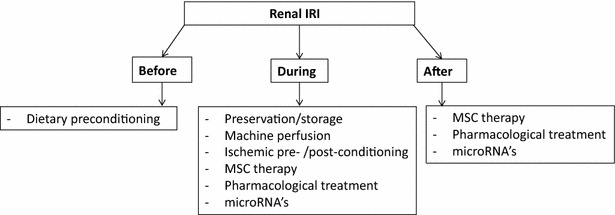
Overview of various therapies before, during and after kidney transplantation, which are capable of ameliorating renal ischemia reperfusion injury in animal models

In this review, we focus on experimental and clinical studies on dietary preconditioning, preservation, ischemic pre- and post-conditioning, cell therapy, pharmacological treatment and microRNAs as intervention strategies to reduce renal IRI. In addition, we review the current state of translation to the clinical setting of these interventions (Fig. 1).

### Dietary preconditioning

Dietary restriction is a reduction in food intake without malnutrition, and is associated with extended life span, improved metabolic fitness and increased resistance to oxidative stress in a wide range of organisms [28–30]. In mice, short-term 30 % dietary restriction or 3 days of fasting, reduced kidney damage and dysfunction and improved survival after renal IRI [30]. Short-term dietary restriction and fasting increased expression of cytoprotective genes and decreased the expression of inflammatory markers [30]. Food restriction leads to a reduction in both calorie and nutrient intake, yet the contribution of calories or nutrients to the protective effect on renal IRI is unknown [28]. Verweij et al. [31] showed that the benefits of preoperative fasting are not affected by the intake of calories via glucose water during fasting from solid food. Subsequently, diets lacking protein or even the essential amino acid tryptophan for 6–14 days resulted in similar protection against renal IRI in mice [32–34]. Therefore, a preoperative calorie restricted diet might be a non-invasive way to reduce IRI after human kidney transplantation. Although the beneficial effects of a preoperative diet are in apparent conflict with the patients’ nutritional wellbeing [35, 36], several recent clinical studies showed that the human response to dietary restriction is similar to that observed in experimental mammalian models [37, 38].

In human living kidney donors short-term dietary restriction before surgery is feasible, well tolerated and safe [39], although the conditions to induce a similar powerful protection against IRI as in mice have not been elucidated yet. More clinical research is needed to translate the beneficial effect of preoperative diets from animals to humans.

### Preservation

Another option to decrease IRI is to minimize damage caused during ischemia. Prolonged cold ischemia time has a strong association with development of DGF [19, 25], but a decrease in cold ischemia time is difficult due to logistics, allocation and organ transport. Optimisation of conditions during cold ischemia time is therefore essential. Cold storage solutions were designed to increase organ tolerance and preserve cellular integrity during ischemia [40, 41]. Reducing the temperature of the kidney to 4 °C reduces enzyme activity, decreases oxygen requirement and lowers metabolism by 58 % [42]. Some studies add nutrients or pharmacologically active agents to the preservation solution (reviewed in [43]). Cold storage is still considered the gold standard in kidney preservation.

During machine perfusion, the organ is attached to a machine during preservation, which pumps preservation solution through the organ. It creates the possibility to maintain hemodynamic stimulation, administer nutrients to the kidney and even eliminate toxins. A large international prospective randomized controlled trial The Netherlands showed the benefits of machine perfusion by reducing the incidence and duration of DGF, in DCD kidneys [44]. Also, machine perfusion of extended criteria donor kidneys reduced the rate of DGF [45, 46], is feasible, and safe [47]. In a meta-analysis, Deng et al. [48] compared the transplant outcomes in patients receiving DCD kidneys preserved by machine perfusion or by static cold storage. Recipients with a DCD kidney preserved by machine perfusion had a decreased incidence of DGF compared to static cold storage. However, there is no significant difference between the two groups in incidence of primary non-function, graft survival or patient survival after 1 year.

Hypothermic machine preservation slows down the metabolism of the kidney and allows an organ to be stored without oxygen for a short period of time but this process also causes cellular damage. Therapeutic agents have been added to the preservation solution during hypothermic machine preservation but the hypothermic conditions make it difficult for the agent while the metabolism is blocked. Maintaining the kidney at a normothermic temperature has many advantages. The kidney is able to regain function and can minimize the cold ischemia time. The kidney can be maintained in a stable state and it provides the opportunity to add therapeutical agents to a functioning organ [49]. Machine perfusion is one of the therapeutic interventions that is making the translation to humans. Randomized controlled trials are now being developed and will guide machine perfusion into the clinical arena.

### Ischemic pre-/post-conditioning

Ischemic conditioning is defined as applying a brief ischemic insult to an organ through brief (repetitive) sequences of ischemia and reperfusion before or after an ischemic attack to provide resistance against IRI. Ischemic preconditioning (IPC) and ischemic post-conditioning (IPoC) were both developed in cardiac research, but may be applied in the kidney as well, reviewed in [50].

### Ischemic pre-conditioning

In 1986, protection against IRI by IPC was first seen in canine hearts [51]. Dogs were preconditioned with four repetitive sequences of ischemia and reperfusion each 5 min, followed by 40 min of occlusion. IPC limited infarct size to 25 % compared to the control group. After these findings many animal experiments have been done to reproduce this protective effect in other organs [52]. In the kidney, IPC induces improved renal function and histology after transplantation [53].

In remote IPC, the ischemic trigger is not applied locally to the target organ, but on another ‘remote’ organ [54]. Patients undergoing elective coronary artery bypass graft surgery underwent remote IPC consisting of three 5-minute cycles of right upper limb ischemia directly after anesthesia. Remote IPC reduced serum troponin-T release compared to patients undergoing coronary artery bypass graft surgery without IPC [55].

In a rat model, renal IRI was induced by a right nephrectomy and clamping the left renal artery for 60 min. Remote IPC was induced by 5-minute cycles of ischemia and reperfusion, occluding the right hind limb. The remote IPC groups showed lower levels of kidney dysfunction and damage [56]. In a renal IRI pig model, remote IPC was induced by clamping the left iliac artery for 10 min, which showed no beneficial effects on renal function or histology [57]. In humans, kidney transplant recipients underwent remote IPC and were compared to paired recipients without IPC. Remote IPC was induced by three cycles of 5 min of brief repetitive ischemia by clamping the exposed external iliac artery. Serum creatinine levels were lower in the remote IPC group, while glomerular filtration rates were higher during the first 14 days post-transplant. These results suggest that remote IPC has beneficial effects on the early recovery of renal function after kidney transplantation [58].

Remote preconditioning is a potential therapeutic strategy that can reduce renal IRI, and is simple to apply, non-invasive and virtually cost-free, but large multi-center clinical trials using remote IPC are needed to improve the level of evidence and implement remote IPC in the clinical setting. Results of a large international prospective randomized controlled trial (CONTEXT trial) are eagerly awaited [59].

### Ischemic post-conditioning

IPoC, defined as rapid, intermittent interruptions of blood flow at the onset of reperfusion can reduce myocardial infarct size in animal models [60, 61]. The use of IPoC in humans undergoing cardiac surgery showed better post-operative outcomes [62]. Similar beneficial effects have also been observed in animal models of renal IRI [63]. IPoC reduced tubular necrosis after reperfusion, and attenuated renal dysfunction [64]. Its observed benefits are associated with an enhanced expression level of SOD and inhibition of apoptosis [65]. These effects are seen in different animal species with different index ischemia times and different algorithms. Only two studies did not observe a significant difference in renal function, which could be explained by the time points of analyzing renal function which were either too early (2 h) or too late (12 weeks) after reperfusion [66, 67].

Contrary to IPoC, remote IPoc has only been performed in two renal IRI rat studies [56, 68]. Remote IPoC of the hind limb resulted in significant improvement in renal function 24 h after IRI. Sequences of ischemia and reperfusion during the ischemic episode, PER-conditioning, was able to reduce renal IRI even further [68]. As with IPC, the first attempts to translate IPoC into human kidney transplantation are already being done [69]. Unfortunately, the robust beneficial effects as seen in animal experiments, have not been observed yet. IPoC is feasible and safe in patients undergoing kidney transplantation, but the proper algorithm that reduces the incidence of DGF still has to be found [69].

### Cellular therapy

Administration of cells to modulate the course of IRI has attracted considerable interest. Two cell types in particular, mesenchymal stem cells (MSC), and regulatory T cells (Tregs) have been investigated.

Mesenchymal stem cells (MSCs) are able to differentiate into cell types other than their tissue of origin, are non-immunogenic, immunosuppressive, able to migrate, secrete growth factors and anti-apoptotic cytokines [70], and might play a role in tissue repair. Due to these characteristics, MSCs are promising as a cell therapy to reduce renal IRI. In rodent renal IRI models, MSCs were able to upregulate the cytoprotective genes HO-1 and SOD [71–73], reduce oxidative stress and apoptosis [71], and improve kidney function [71, 72, 74]. Furthermore, kidneys treated with MSCs showed a stronger regenerative response [75].

Subsequently, in large-animal models, MSCs failed to reduce cell death and no changes in proliferation or cytokine release were found [76, 77]. It might be that the optimal time window for stem cell therapy is different in large-animal models than in rodents. Another problem is poor cell survival of injected MSCs. After intravenous injection MSCs home to the lungs and within 24 h the majority of MSCs die, MSCs do not migrate to the site of injury and do not contribute to structural renal repair [78]. This suggests that the effect of the MSCs might result from paracrine or endocrine effects unrelated to their differentiation capacity [78–82].

Early clinical trials have attempted to translate the potential immunosuppressive effects of MSCs, but results were not convincing. Perico et al. [83] were the first to report on two patients undergoing living kidney transplantation and receiving an infusion of autologous MSCs on post-transplant day 7. Serum creatinine levels were increased in MSC-treated patients 7–14 days after infusion, suggesting dysfunction of the graft. 1 year post-transplantation kidney biopsies showed no signs of rejection. Their conclusion was that MSC therapy in kidney transplantation is feasible, although timing, doses and immunosuppressive medication may need to be adapted for optimal effect. Reinders et al. [84] studied the feasibility of autologous MSC administration in kidney transplantation recipients and showed it to be feasible and safe, although the study does not allow conclusions on efficacy. Peng et al. [85] combined MSCs with a sparing dose of Tacrolimus (50 % of standard dose) in living-related kidney transplant recipients. Patients received two infusions of MSCs, the first directly into the renal artery at the time of transplantation, the second intravenously 1 month later. Results suggest that MSC therapy is safe and could reduce the dosage of Tacrolimus. The results of both animal models and clinical trials are encouraging, but the low number of randomized controlled trials and small numbers of patients make it difficult to draw definitive conclusions and implement MSC therapy in transplantation [86].

The knowledge that Tregs have a crucial role in control of autoimmunity and tolerance induction in transplantation has made the induction of-, or infusion of Tregs a possible treatment for an array of inflammatory conditions, IRI. In humans, intravenous infusion of Tregs is not only feasible and safe, but reduced the incidence of graft versus host disease in patients with hematologic malignancy that were treated with stem cell transplantation [87].

Furthermore, after inducing renal IRI in rodents, Tregs are able to suppress renal inflammation and preserve renal function [88]. In a mouse model, Treg deficiency resulted in enhanced renal inflammation, acute tubular necrosis and loss of function. Suppletion of Tregs protected mice from renal dysfunction and improved survival [89]. Although the use of Tregs as a cellular therapy against renal IRI seems promising, studies in humans with renal IRI are lacking. The use of Tregs in humans is troubled by numerous challenges. The dose of Tregs needed for therapeutic efficacy is unclear, the isolation of pure Tregs is difficult due to the absence of Treg-specific cell surface markers and safety is still a topic of concern [90].

### Pharmacological treatment

Although many pharmacological agents are effective in experimental models of IRI and acute kidney injury, none of these have successfully been implemented in standard clinical care protocols. With few exceptions, most do not enter the clinic. An overview of tested pharmacological substances is given by Bajwa et al. [91]. Of the eight substances that might reduce inflammation and reduce cytotoxicity they focused on, only two were tested in clinical studies for acute kidney injury (statins and erythropoietin). However, in human studies the results on renal IRI induced acute kidney injury are conflicting. Retrospective case controlled studies found that statins reduced acute kidney injury in patients with contrast-induced nephropathy [92, 93], whereas a prospective study did not find any beneficial effects [94]. Remarkably, a number of observational studies suggested that in the first few weeks and months of starting a statin, statins were associated with the early development of acute kidney [95]. Due to the adverse data and the lack of good prospective randomized controlled trials, there is no evidence that statins reduce the incidence of acute kidney injury.

Preconditioning with erythropoietin protects against IRI in rodents [96, 97]. Encouraged by these results erythropoietin was injected intravenously in humans before surgery, and was able to reduce the incidence of acute kidney injury in patients who underwent a coronary artery bypass [98]. Xin et al. [99] published a meta-analysis including four randomized controlled trials that investigated high-dose erythropoietin on graft function after kidney transplantation. The results showed that high-dose erythropoietin is able to reduce the number of patients with DGF, but these results did not reach significance. However, Vlachopanos et al. [100] published a meta-analysis to explore the impact of recombinant human erythropoietin on DGF in kidneys from deceased donors. Four randomized controlled trials were included and perioperative high-dose recombinant human erythropoietin was compared with placebo or no therapy. High-dose recombinant human erythropoietin did not affect mortality, acute rejection, DGF or kidney function 4 weeks after transplantation. Remarkably, the systolic blood pressure was significantly higher in patients treated with recombinant human erythropoietin. These results question the efficacy and safety of high-dose human recombinant erythropoietin in humans. Despite the promising results in animal models, translating these findings to the clinic is difficult. Variable factors as dosage and time points of injection might be a topic of interest for further clinical trials.

Recently, nonerythropoietic peptides derived from the three-dimensional structure of erythropoietin were shown to exert tissue protective properties. It was shown that the helix B surface peptide of erythropoietin is responsible for the tissue protective effect of erythropoietin and has a much better stability [101]. In a mouse renal IRI model, helix B peptide improved renal function, decreased apoptosis, inflammation and histological injury [102]. Yang et al. [103] added the helix B peptide to preservation and reperfusion solutions used to normothermically perfuse porcine kidneys after 20 min of warm and 18 h of cold ischemia. Adding helix B peptide to the reperfusion solution improved the renal blood flow, oxygen consumption and urine output during reperfusion and decreased renal tissue damage. Helix B peptide could be the key needed to translate the beneficial effects of erythropoietin to human transplantation.

### MicroRNA’s

MicroRNA’s are RNA-molecules of 20–25 nucleotides long. They are capable to inhibit protein transcription by stimulating degradation of mRNA [104]. The majority of gene expression is regulated in this way. A promising quality of microRNA’s is their stability in body fluids [105], which makes them a good candidate to act as a biomarker or as a therapeutical target.

One microRNA can inhibit more than 100 genes, so determining the role of microRNAs in IRI is difficult. The few studies on microRNAs in renal IRI failed to elucidate an unequivocal microRNA-signature [106–108]. Expression profiling of microRNAs following renal IRI in a mouse model showed that nine miRNAs (miR-21, miR-20a, miR-146a, miR-199a-3p, miR-214, miR-192, miR-187, miR-805, and miR-194) are differently expressed compared to sham animals [108]. In vitro studies revealed that miR-21 is expressed in proliferating tubular epithelial cells, and overexpression of miR-21 has a protective effect against cell death. This might suggest that miR-21 plays a role in protection against IRI. In humans, microRNA expression profiles have been analysed to see if microRNAs may predict the outcome after kidney transplantation [106, 107]. In renal biopsies of patients with acute rejection, 20 differentially expressed miRNAs were identified [106]. These expression profiles may provide useful information about the outcome after kidney transplantation. Unfortunately, research so far has not brought major insights in the role of microRNAs as therapeutic target or agent in both animals and humans [109].

## Discussion

The improved results after kidney transplantation and the increased waiting list have contributed to the growing gap between organ demand and supply. Extension of the donor pool is needed to diminish this gap. Therefore, there has been a shift to the use of DCD donors and extended criteria donors. DCD donors have an increased incidence of DGF compared to DBD donors, while the rate of acute rejection is similar in both groups [9, 10]. Kidneys from extended criteria donors have a higher risk of DGF, lower graft survival and suboptimal kidney function [13, 14]. Although renal IRI is inevitable during transplantation and has detrimental effects on the outcome, there is no therapy available. Therefore, finding a method to ameliorate renal IRI is of major interest. Renal IRI can be treated before-, during-, and after transplantation, or a combination thereof. When treatment is applied before ischemia, translation of these data is difficult since in the human setting, treatment before ischemia would imply treatment of the donor. This raises ethical concerns in DCD donors [110]. During treatment of the living donors, the donor must give full consent and treatment should not interfere with the donor’s health. An option for treatment of the (living) donor before transplantation is dietary preconditioning. Van Ginhoven et al. [39] showed that dietary pretreatment of living donors is feasible and safe, but the robust effects on reducing IRI as observed in mice are lacking. Despite many experimental studies which show beneficial effects on an array of treatments and interventions against IRI, translation to humans has not been successful [111]. In animal experiments, genetic variability is low and mostly young, healthy, males are used. This is obviously not representative for the population that is undergoing kidney transplantation and is experiencing renal IRI. Overweight, comorbidities, old age, gender and the use of medication can all interfere with the effects of studied methods to ameliorate renal IRI [112, 113]. Another limitation of animal experiments may be the use of warm ischemia models to mimic transplantation induced IRI [30, 31, 71, 74]. The use of cellular therapy is difficult to translate to humans due to the differences between animals and humans. More clinical trials are needed to evaluate the effect of both MSC and Tregs. It would be of tremendous value to use MSCs, or to be able to induce the production of Tregs in the recipient to ameliorate renal IRI. Besides these translational difficulties, another problem in the treatment of IRI is its pathophysiological complexity. Many pathological mechanisms contribute to IRI and can be focused on. Studies on IRI treatment are therefore divers, and the probability to find a single therapeutical agent is low. Besides that, experimental therapeutical agents may induce adverse side effects [114–117] or be carcinogenic [118, 119] which limits their use in humans. Another difficulty in translating animal results into humans is the publication bias. It is difficult to get an objective overview of the results of experimental therapies since there may be a bias towards publication of studies with a positive outcome [120]. Nevertheless, machine perfusion and (remote) ischemic pre- and post-conditioning are promising treatment options, which are feasible and safe. Especially machine perfusion induces beneficial effects on kidney function after transplantation in various donor types, and large randomized controlled trials are being conducted. The use of machine perfusion is actually making the translation to the clinical arena.

## Conclusion

Renal IRI is a highly relevant detrimental consequence of kidney transplantation and therefore an important topic in transplantation research. Studying renal IRI is complex though, coping with translational difficulties, and multifactorial pathophysiological mechanisms. Although animal studies have resulted in promising methods to ameliorate renal IRI, we are still lost in translation since only few animal data are finding their way into the clinic and improve transplant outcome. This gap in our understanding of IRI may be filled in the next years with new data derived from more sophisticated animal models and results of large randomized controlled trials.

## Abbreviations

IRI: ischemia reperfusion injury
DGF: delayed graft function
DBD: donation after brain death
DCD: donation after circulatory death
MSCs: mesenchymal stem cells
IPC: ischemic preconditioning
IPoC: ischemic post-conditioning
